# Macrophage Infiltration Induces Gastric Cancer Invasiveness by Activating the β-Catenin Pathway

**DOI:** 10.1371/journal.pone.0134122

**Published:** 2015-07-30

**Authors:** Ming-Hsun Wu, Wei-Jiunn Lee, Kuo-Tai Hua, Min-Liang Kuo, Ming-Tsan Lin

**Affiliations:** 1 Department of Surgery, National Taiwan University Hospital, Taipei, Taiwan; 2 Department of Medical Education and Research, Wan Fang Hospital, Taipei Medical University, Taipei, Taiwan; 3 Institute of Toxicology, College of Medicine, National Taiwan University, Taipei, Taiwan; 4 Department of Medical Education & Bioethics, Graduate Institute of Medical Education & Bioethics, National Taiwan University College of Medicine, Taiwan; Istituto Superiore di Sanità, ITALY

## Abstract

**Background:**

Despite evidence that activated macrophages act in an inflammatory microenvironment to promote gastric tumorigenesis via β-catenin signaling, the effects of β-catenin signaling on gastric cancer cell metastasis and the relationship of these cells with surrounding tumor associated macrophages have not been directly studied.

**Methods:**

Immunohistochemical staining was employed to analyze 103 patients. An invasion assay was used to evaluate the relationship between macrophages and gastric cancer cells. β-catenin gain-of-function and loss-of-function approaches were performed. To assess the β-catenin regulation mechanism in gastric cancer cells, Western blotting and reverse-transcription polymerase chain reaction were used.

**Results:**

Increased density of macrophages was associated with advanced stage and poor survival. Gastric cancer cell lines co-cultured with macrophages conditioned medium showed increased nuclear accumulation of β-catenin and increased invading ability. AKT but not ERK regulated β-catenin translocation. MMP7 and CD44, both β-catenin downstream genes, were involved in macrophage-activated gastric cancer cell invasion.

**Conclusion(s):**

Collectively, the clinical data suggest that macrophage infiltration is correlated with increased grade and poor prognosis for gastric cancer patients who underwent radical resection. Macrophages may induce invasiveness by activating the β-catenin pathway.

## Introduction

Gastric cancer (GC) is among the most common cancers worldwide and almost two-thirds of such patients will die of their disease.[1] GC is closely associated with *Helicobacter pylori* infection, which leads to chronic inflammation.[2] This inflammatory microenvironment is characterized by the presence of host leukocytes with mainly macrophages in both the supporting stroma and tumor tissues.[3] Researches indicate that tumor-associated inflammatory responses, both local and systemic, are important independent factors in tumor progression and metastasis.[4, 5] However, the links between these molecular mediators and chronic inflammation are not fully understood.

Activation of the Wnt pathway is an important step in carcinogenesis. Mutations along the Wnt–β-catenin pathway occur in approximately 90% of colorectal and hepatocellular carcinomas and in about 30% of GCs.[6, 7] In addition, tumor necrosis factor-α (TNF-α) derived from activated macrophages promotes β-catenin activity in GC cells.[8, 9] However, despite evidence that activated macrophages act in an inflammatory microenvironment to promote gastric tumorigenesis via β-catenin signaling, the effects of activated β-catenin signaling on GC cell metastasis and the relationship of these cells with surrounding tumor associated macrophages (TAMs) have not been directly studied.

Based on above findings, we hypothesized that the β-catenin pathway may also be involved in regulating macrophage-induced GC metastasis. In this study, we first examined the density of infiltrated macrophages and the expression of β-catenin in gastric carcinoma tissues using immunohistochemistry (IHC). The clinicopathological characteristics of gastric carcinoma and prognosis were demonstrated. Furthermore, we found that macrophages induce nuclear translocation of β-catenin and enhance the invasion ability of GC cells. Our findings demonstrate and further support an important link between TAM and its downstream signaling mediator, β-catenin, in regulating GC metastasis.

## Material and Methods

### Patients and Specimens

The study was approved by the Institutional Review Board and Human Ethics Committee of National Taiwan University Hospital. Written consent for using the samples for research purposes was obtained from all patients prior to surgery. Patient information was anonymized and de-identified prior to analysis.

The study retrospectively enrolled 205 patients diagnosed with GC who received radical surgical resection at the Department of General Surgery, National Taiwan University from January 1998 to January 2002. Of these, 102 patients who lacked follow-up data or who died from perioperative complications were excluded and the remaining 103 patients were included in analyses. Clinicopathological variables were classified according to TNM classification (5th edition, 1997) and the characteristics are summarized in Table 1. Overall survival (OS) was defined as the interval between surgery and death or between surgery and the last follow up for surviving patients.

**Table 1:** Associations between intratumoral CD68-positive and patients' clionicaopathologic characteristics.

	Low CD-68 positive (<671)	High CD-68 positive (> = 671)	*p*
Number of patients	63	40	
Gender			
Male	39	21	0.346
Female	24	19	
Age (yr)	58.97+-13.77	63.40+-13.55	0.112
Histilogical classification			
Intestinal type	28	22	0.493
Diffuse type	35	18	
Mixed type	3	3	
Tumor depth			
pT1-3	56	25	0.001
pT4	7	15	
Lymph node metastassis			
pN0-1	50	26	0.106
pN2-3	13	14	
Distant metastasis			
M0	62	39	0.744
M1	1	1	
Stage			
S1-3	52	26	0.043
S4	11	14	

### Monoclonal Antibodies and IHC

The resected specimens of GC were fixed in formalin and embedded in paraffin. Sections were examined for TAM infiltration and β-catenin using monoclonal anti-CD68 antibody (clone KP1, DakoCytomation, Glostrup, Denmark) and monoclonal β-catenin antibody (clone 15B8, Sigma-Aldrich, St. Louis, MO), respectively.

### Evaluation of Immunostaining

To evaluate the density of tissue-infiltrating CD68+ macrophages, tissue sections were screened by a board certified pathologist (CI Jan) at low power (100×) and the five most representative fields were selected at 400× magnification. The results were counted manually and were expressed as the sum of the number of cells in five 400× microscopic fields for each area of every specimen.

### Cell Culture

GC cells AGS, N87 (purchased from the American Type Culture Collection (Manassas, VA)), MKN45 (purchased from the Health Science Research Resources Bank (Osaka, Japan)) and TSGH (purchased from the Bioresource Collection and Research Center (Hsinchu, Taiwan)) and human monocytic cell line THP-1 (purchased from American Type Culture Collection (Manassas, VA)) cells were grown in RPMI-1640 medium (Life Technologies, Inc., Rockville, MD) with 10% fetal bovine serum (FBS, Life Technologies, Inc.) and 2 mM L-glutamine (Life Technologies, Inc.).

THP-1 cells were seeded into culture dishes and induced to differentiate into macrophages by incubation with 100 ng/ml 12-O-tetradecanoylphorbol-13-acetate (TPA, Sigma-Aldrich) for 24 h. The macrophages were washed three times with RPMI medium containing 10% FBS, incubated in this medium for another 24 h to eliminate the effect of TPA and incubated in serum-free media for another 24 h. The harvested and pooled culture supernatants were used as macrophage conditioned medium (CM) as previously described.[5, 10]

### Proliferation/Viability Assay

GC cells were seeded in 96-well plates and co-cultured with or without macrophage CM. The proliferation/viability was determined by a colorimetric assay using 3-(4,5-dimethylthiazol-2-yl)-2,5-diphenyltetrazolium bromide (MTT) (Sigma-Aldrich).

### In Vitro Invasion Assay

The invasion assay was conducted using transwell cell culture chambers (Millipore Corp., Billerica, MA). The invading cells were counted at 400× magnification in 10 different fields for each insert. The experiment was repeated three times.

### RNA Extraction and RT-PCR

After stimulation with or without macrophage CM for 6 h, GC cells were collected and total RNA extracted using the Trizol reagent kit (Invitrogen, Carlsbad, CA) following the manufacturer’s instructions. cDNA was amplified by polymerase chain reaction (PCR) using primers for MMP7 (forward: 5’- AGTCAATCCTGCCTTCTTAGCC, reverse: 5’- GCTCCCACCTTCCCTCTTGG), CD44 (forward: 5’- TGCCGCTTTGCAGGTGTAT, reverse: 5’- TCCCATGTGAGTGTCTGGTAGC), cyclin D1 (forward: 5’- CCCAGCCATGGAACACCA, reverse: 5’- GGAGGGCGGATTGGAAATGA), c-myc (forward: 5’- AAAGAAGGTGTTGGGGTCGG, reverse: 5’- TGGCTCCCCCTGTTATTTGG) and GAPDH (forward: 5’- GGGTGATGCAGGTGCTACTT, reverse: 5’- GGCAGGTTTCTCAAGACGGA).

### Nuclear and Cytoplasmic Fractionation

After stimulation with or without macrophage CM for 6 hours, GC cells were lysed in 300 μl lysis buffer on ice and centrifuge at 5,400 rpm. Supernatant was collected as a cytosolic fraction. Nuclear fractions were collected, then run in sodium dodecyl sulfate polyacrylamide gel electrophoresis (SDS-PAGE) for Western blot analysis.

### Western Blotting Analysis

After stimulation with macrophage CM, cells were washed with PBS, scraped into RIPA buffer and centrifuged. The cell lysates were subjected to 10% SDS-PAGE and transferred to a polyvinylidene fluoride (PVDF) membrane (Millipore Corp.) The membrane was probed using primary antibodies for β-catenin, AKT, ERK, JNK (SC1496, SC8312, SC154, SC474, Santa Cruz Laboratories, Santa Cruz, CA, USA), MMP7 (MAB3322, CHEMICON, Temecula, CA, USA), CD44 (ab51037, Abcam, Cambridge, MA, USA), c-myc (GTX103436, GeneTex, Hsinchu City, TAIWAN), cyclin D1 (SC246, Santa Cruz Laboratories, Santa Cruz, CA), β-actin (ab8226, Abcam), HDAC1, p-AKT, p-ERK, p-JNK (SC7872, SC7985-R, SC7383, SC6254, Santa Cruz Laboratories, Santa Cruz, CA) and secondary antibody (Santa Cruz Biotechnology).

### Immunocytochemistry

Anti-total β-catenin antibody was used as the primary antibody, and anti-mouse Immunoglobulin G (IgG) Alexa 594 was used as the secondary antibody. Cover slips were then mounted using mounting medium containing DAPI for nuclear staining.

### Lentiviral production and infection

Short hairpin RNAs (shRNAs) were purchased from the National RNAi core Facility at Academic Sinica (Taipei, Taiwan). The target sequence of β-catenin shRNA 1 was 5’- CCGGTCTAACCTCACTTGCAATAATCTCGAGATTATTGCAAGTGAGGTTAGATTTTTG-3’; that of β-catenin shRNA 2 was 5’-CCGGTTGTTATCAGAGGACT-AAATACTCGAGTATTTAGTCCTCTGATAACAATTTTTG-3’. The lentiviral vector and its packaging vectors were transfected into 293T packaging cells by calcium phosphate transfection. Briefly, 293T cells were split (10^6^) into 10 cm^2^ dishes 1 day before transfection. Then, cells were transfected with 10 μg shRNA vectors and 10 μg of pCMVΔR8.91 (the packaging vector) and 1 μg of pMD.G (the envelope vector). After 5 h of incubation, transfection medium was replaced with fresh culture medium. Forty-eight hours later, lentivirus-containing medium was collected from transfection and spun down at 1500 rpm for 5 min to pellet the cell debris, the supernatant was filtered through a 0.45-μm filter, and target cells were infected with fresh lentivirus-containing medium (supplemented with 8 μg/ml polybrene) for 24 h.

### Statistical Analysis

Statistical analysis of the clinical features was done using the χ^2^ test and the degree of infiltration between the two groups was compared using t-test. Survival curves were constructed using the Kaplan-Meier method and statistical significance was analyzed using the log-rank test. A p-value of less than 0.05 was considered statistically significant.

## Results

### Correlation of Immunohistochemical Variables with Clinicopathologic Features in GC Patients

**Fig 1:**
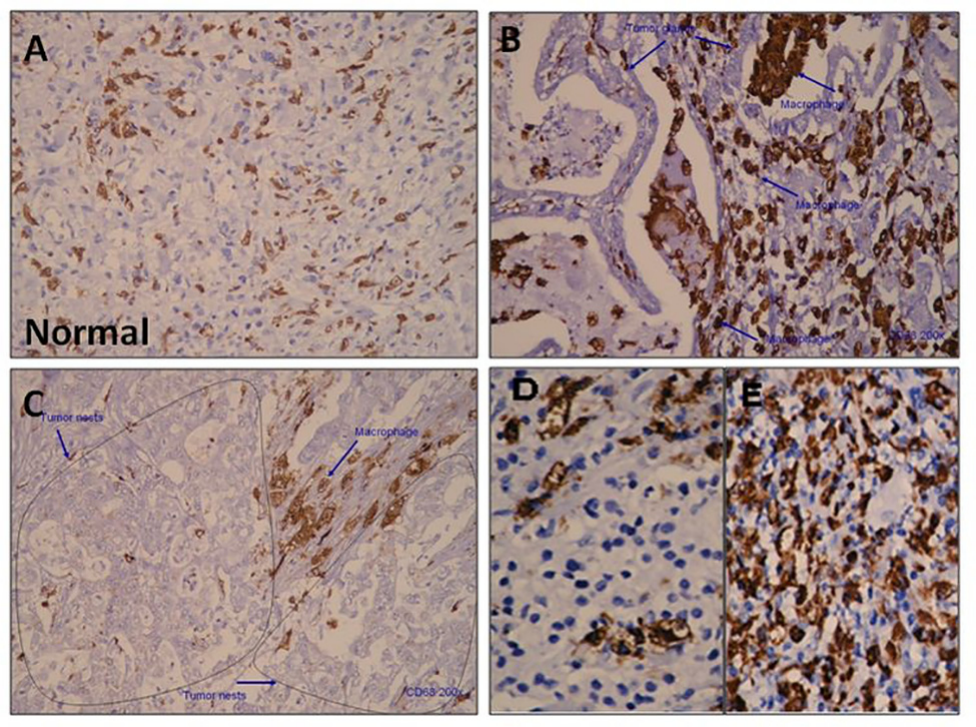
Immunohistochemistry analysis. Immunostaining against CD68 was performed on tissue slides from gastric cancer patients. CD68+ macrophage staining was sparse in the normal gastric mucosa (A). CD68+ cells were detected in both the stroma and tumor nest (B & C). The density of CD68+ macrophages in pathological tumor staging (pT) 1 (D) and pT4 (E) tumor lesions.

**Fig 2:**
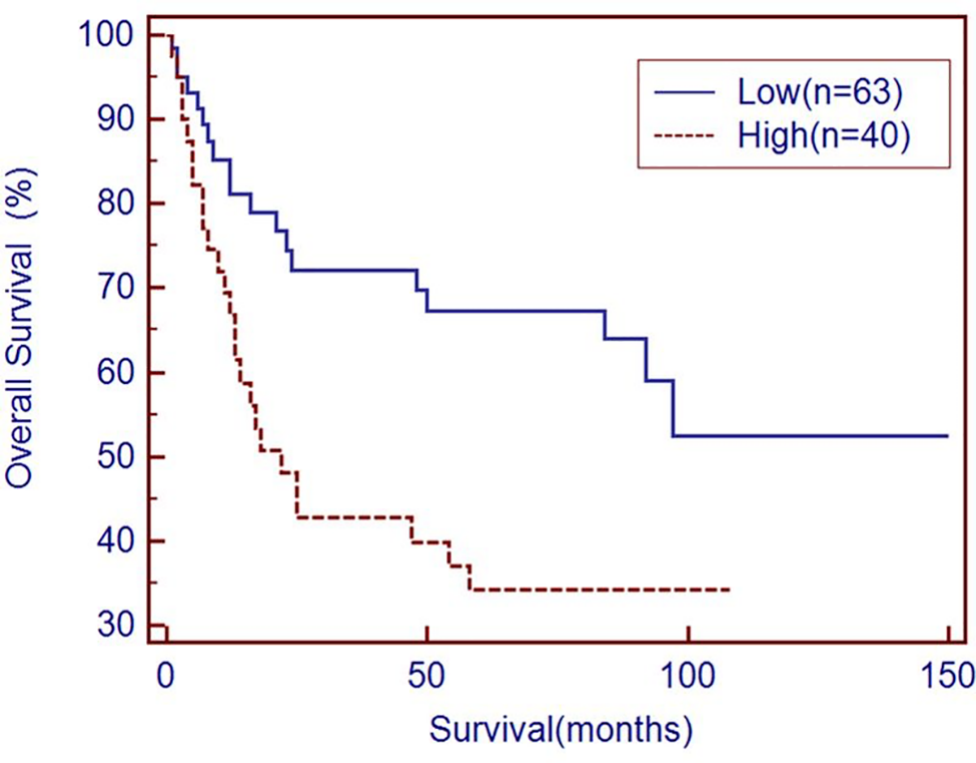
Kaplan-Meier overall survival curves for gastric cancer patients as TAM density (Low: TAM<671, blue line; High: TAM>671, brown line; *p* = 0.0073).

Immunohistochemical analysis showed a cytoplasmic CD68 staining pattern for macrophages. CD68+ macrophages were distributed throughout peritumoral and intratumoral tissues but only sparsely scattered in the normal mucosa (Fig 1A–1C). The density of CD68+ macrophages was especially high in pathological tumor stage 4 (pT4) tumor lesions compared with pT1 lesions (Fig 1D). To determine the association between clinical characteristics and CD68+ macrophage density, counts of CD68+ macrophages were divided into those above and below the median values. The number of CD68+ macrophages was found to be associated with tumor depth and stage (p = 0.001 and p = 0.043, respectively) (Table 1). This observation suggests that CD68+ macrophages may be important in promoting tumor invasion. For further analysis, Kaplan-Meier survival curves were plotted to determine the association of macrophages with survival (Fig 2). The density of CD68+ macrophages in the tumor tissue was negatively associated with overall survival (p = 0.0073). Patients with high numbers of tumoral CD68+ macrophages had shorter overall survival than those with low numbers of CD68+ macrophages.

**Fig 3:**
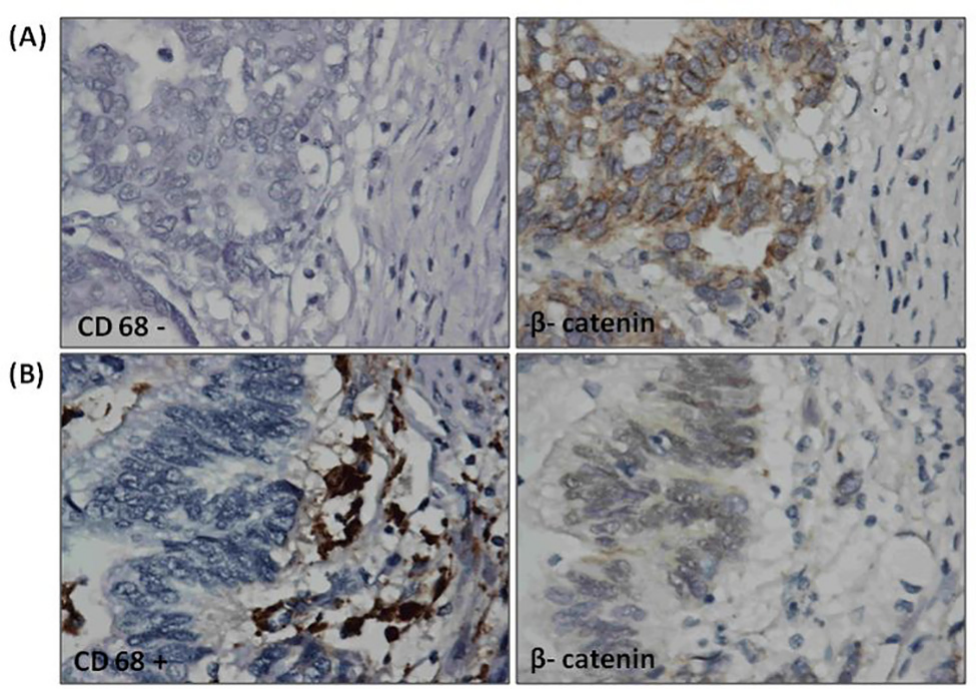
Immunohistochemistry analysis. β-catenin was highly expressed in tumors. (A) In most cases, β-catenin expression was primarily located in the cytoplasm. However, (B) strong nuclear staining of β-catenin in GC cells was positively associated with CD68+ macrophages in the tumor lesions.

Increased expression and stronger signal of β-catenin were observed by IHC staining in tumors. In most cases, β-catenin expression was primarily located in the cytoplasm (Fig 3A). Interestingly, strong nuclear staining of β-catenin GC cells was associated with CD68+ macrophages in the tumor lesions, especially in front of the tumor invasion (Fig 3B). On the basis of these results, we hypothesized that macrophage infiltration may activate β-catenin signaling in gastric tumor cells, which then likely leads to invasion in the gastric malignancy.

### Requirement of Macrophages for Tumor Invasion in GC and Activated Macrophage-Induced Invasion of GC Cells

**Fig 4:**
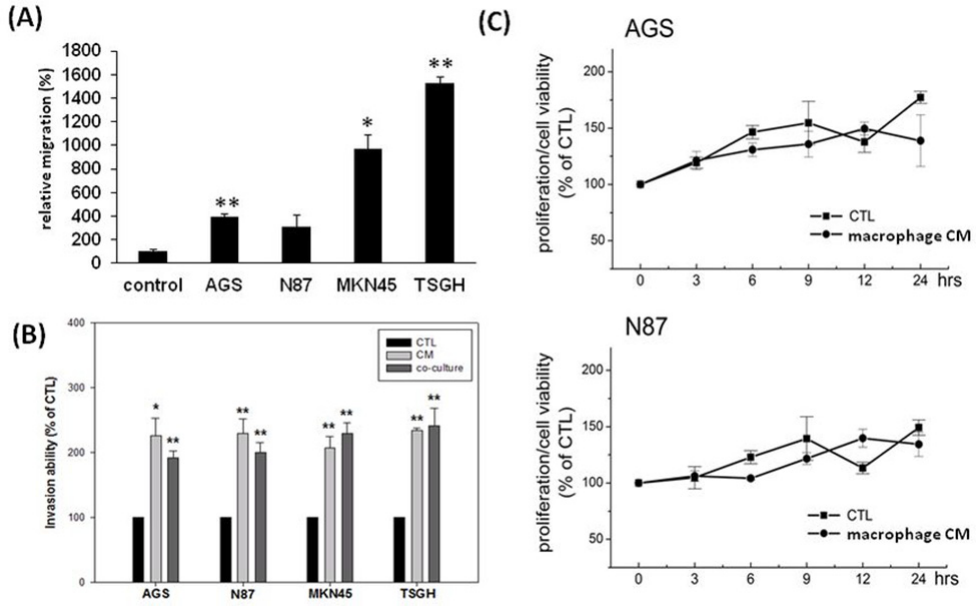
(A) Migration activity of THP-1 monocyte co-cultured with different gastric cancer cell lines (AGS, N87, MKN-45 and TSGH). THP-1 monocyte will be recruited by different gastric cancer cell lines and the recruiting ability is dependent on the degree of malignancy. (B) Invasion ability of GC cells treated by macrophage CM. Four different types of GC cells (AGS, MKN45, N87 and TSGH) were seeded in a Boyden chamber and co-cultured with or without macrophage cells or macrophage CM for 24 hours. Invasion abilities of each cell line were measured *in vitro* for 24 hours. All data represent the arithmetic mean ± SEM. * p < 0.05, ** p < 0.01. (C) Effect of macrophage CM co-cultured with cells. N87 and AGS cells were co-cultured with macrophage CM respectively for 24 hours and cell viability was determined by MTT assay.

To further confirm our clinical findings *in vitro*, we first co-cultured macrophages with different GC cell lines (AGS, N87, MKN-45 and TSGH). All GC cell lines tested would recruit macrophages to migrate and the macrophage-recruiting ability was dependent on the degree of malignancy of the cancer cells as determined by their invasiveness (Fig 4A).[11] To evaluate whether invasiveness or proliferation of GC cells can be regulated by macrophages, AGS, N87, MKN45 and TSGH were co-cultured with and without macrophages or CM from macrophage for 24 h and their invasion and proliferation abilities tested. All cell types co-cultured with macrophage or CM from macrophage showed an near 2-fold increase in the number of invading cells (compared with negative controls, p < 0.05), which is not associated with their proliferation ability (Fig 4B–4D).

### Nuclear Translocation of β-catenin in GC cells by Activated Macrophages

**Fig 5:**
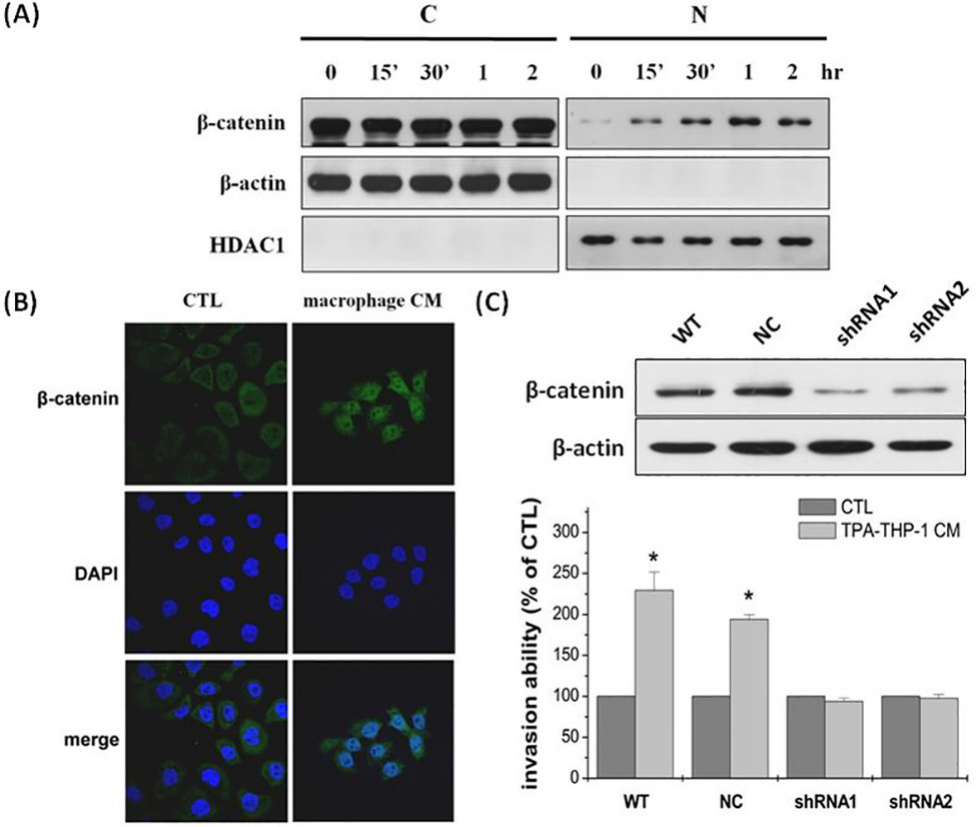
Effect of macrophage CM on β-catenin pathway. (A) β-catenin accumulates in nucleus after treatment with macrophage CM for 30 minutes in N87 cells. (B) Immunofiuorescent images were observed with a confocal microscope. β-catenin positive cells were analyzed by using an anti-β-catenin antibody, which is recognized by secondary rabbit antibody conjugated with FITC, depicted by green fluorescence; Nuclear staining was detected by counterstaining cells with 4', 6-Diamidino-2-phenylindole (DAPI), represented as blue fluorescence. Co-culturing with macrophage CM, immunofiuorescence staining showed β-catenin-FITC complexes translocated into nucleus. (C) Invasion ability of GC cells treated by macrophage CM.

We conducted an experiment in vitro to understand whether β-catenin signaling was involved in macrophage-activated GC cell invasion. As shown in Fig 5A, β-catenin immunoreactivity was found in the nuclear fraction of N87 cells as early as 15 minutes after macrophage CM treatment, and the amount of nuclear β-catenin protein increased in a time-dependent manner. In line with these findings, immunofluorescence showed accumulation and nuclear localization of β-catenin in GC cells co-cultured with macrophage CM as compared to controls cocultured in N87 (Fig 5B). These results strongly suggest that the soluble factors derived from macrophages help mediate the accumulation and nuclear translocation of β-catenin in GC cells. We transiently knocked down β-catenin by shRNA and found decreased β-catenin protein expression within 24 h. Genetic ablation of β-catenin in N87 cells fails to increase their invasive ability after macrophage CM treatment. By contrast, non-transfected and control shRNA had no change in the invasive capability in N87 cells under macrophage CM treatment (Fig 5C). The amount of nuclear β-catenin protein was also increased in the AGS and MKN45 cells after macrophage CM treatment (S1 Fig).

### Effects of Macrophage on Expression of β-catenin and the Downstream Genes in N87 Cells

**Fig 6:**
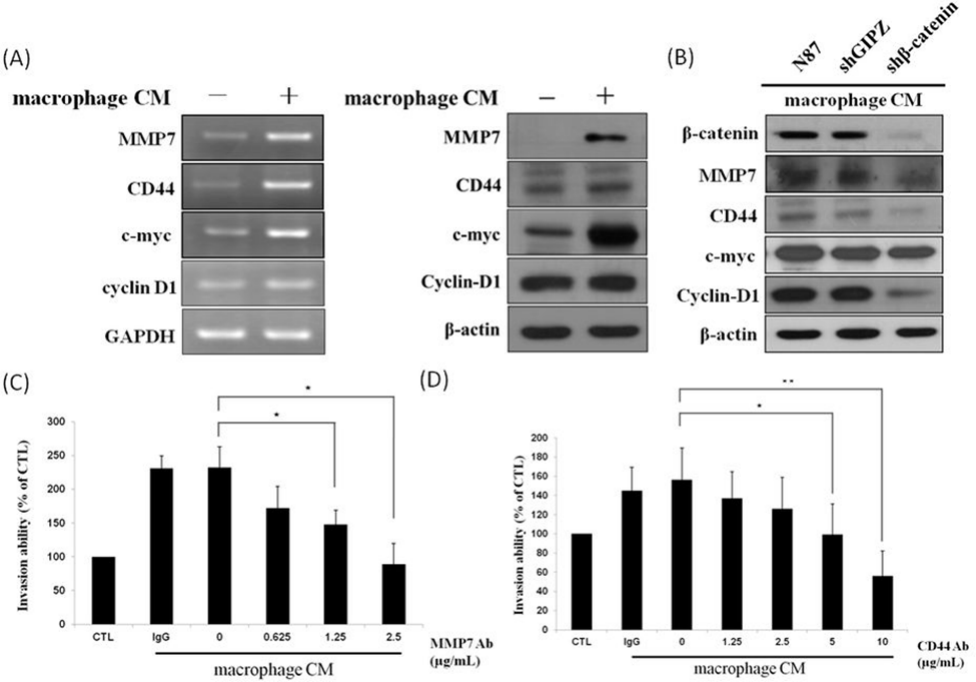
(A) Effect of macrophage CM on mRNA and protein expression of β-catenin down-stream genes. N87 cells were cultured in the presence or absence of macrophage CM. For RNA and protein level, cells were collected after 6 hours and 24 hour treatment respectively. (B) N87 cells with or without knock-down of β-catenin utilizing shRNA-2 were co-treated in the presence or absence of macrophage CM. N87 cells were collected after 24 hours treatment and analyzed with western blot. (C) Effect of MMP7 neutralized antibody on GC cells invasion. N87 cells in the presence of macrophage CM for 24 hours were pre-treated with various doses MMP7 neutralized antibody (0, 0.625, 1.25 and 2.5μg/ml) for 1 hour Isotype IgG was used as a blocking control. (D) Effect of CD44 neutralized antibody on GC cell invasion. N87 cells in the presence of macrophage CM for 24 hours were pre-treated with various doses CD44 neutralized antibody (0, 1.25, 2.5, 5 and 10 μg/ml) for 1 hour. Isotype IgG was used as a blocking control. All data represent the arithmetic mean ± SEM. * *p* < 0.05.

We evaluated the traditional Wnt/β-catenin downstream genes MMP7, CD44, c-myc and cyclin D1 to determinate if they are activated by macrophages. The mRNA and protein levels of MMP7, CD44 and c-myc genes were all significantly increased in N87 cells incubated with macrophage CM and the levels of cyclin D1 increased slightly (Fig 6A). After prompting decreased invasion activity from knocking down β-catenin, MMP7, CD44 and cyclin D1 but not c-myc showed protein down-regulation (Fig 6B). To confirm the relationship between invasion ability and MMP7 and CD44, we tested the ability of a neutralizing antibody to block MMP7 and CD44 activity. Invasion ability was significantly compromised by pretreatment with 2.5 μg/ml anti-MMP7 and with 10 μg/ml anti-CD44 (Fig 6C) respectively, suggesting the involvement of both genes in macrophage-activated GC cell invasion. We found the protein levels of CD44 and cyclin D1 genes were increased in the other GC cell lines (AGS and MKN45) incubated with macrophage CM but MMP7 and c-myc were not significant changed (S2 Fig).

Others have reported a possible intersection of the MAPK pathway with the Wnt/β-catenin signaling pathway.[12, 13] We therefore performed a time course study and found that only phospho-AKT and phospho-ERK expression were upregulated when treated with macrophage CM (S3A Fig). To confirm the interaction between the AKT/ERK pathway and β-catenin, we pretreated cells with either the ERK inhibitor (U0126) or the AKT inhibitor (LY294002). Decrease of β-catenin translocation into the nucleus was only found in cells treated with the AKT inhibitor but not ERK inhibitor, indicating macrophage-avtivated Wnt/β—catenin signaling pathway might be regulated by AKT (S3B Fig). In GC cells, the AKT effect appears to be dominant in the macrophage activated Wnt/β-catenin signaling pathway. Previous studies also suggested that TNF-α produced by macrophages is a key mediator for inflammation and might promote β-catenin activity in tumor cells. We found that utilizing neutralizing antibody against TNF-α in N87 suppress β-catenin activation under exposure to macrophage CM (S4 Fig).

## Discussion

The microenvironment of a tumor is of critical importance in determining leukocyte function and phenotype. Conflicting data has shown antitumorigenic or protumorigenic functions on macrophages in particular, however our data indicates macrophages play a protumorigenic role in GC patients. In the majority of tumors, TAMs produce a myriad of factors that promote tumor growth and angiogenesis.[14, 15] The degree of macrophage infiltration into the cancer cell nest is also a significant predictor of survival in GC patients.[16–18] IHC findings in this study clearly demonstrate that the density of macrophages in GC tissue is correlated with the degree of clinical stage (especially in the tumor [T] stage) and survival in patients. The current studies also imply that macrophages may play harmful roles in advanced GC. This finding is in harmony with studies that suggest that the immune microenvironment may be protumoral rather than antitumoral, despite some conflicting data.[19, 20]

Nuclear accumulation of β-catenin has been reported in approximately one-third of gastric adenocarcinomas, both intestinal and diffuse types.[18] β-catenin is a 92 kDa protein which functions directly in cell-to-cell adhesion.[21] Mutations of β-catenin and aberrant expression of its protein have been implicated in tumor invasion and metastasis.[22, 23] In our IHC findings, β-catenin was located in the nucleus at the invasive front of tumor cells, but not in the most central area of the primary tumors, where it localizes to the plasma membrane. Such observations can also be found in colorectal tumor specimens.[24] Wnt/β-catenin activation in cancer cells located at the invasive front has been postulated as an important step in tumor growth and progression.[25]

Interestingly, we also found β-catenin located in the nucleus of tumor cells with macrophages located adjacently, suggesting a possible relationship between macrophages and the nuclear translocation of β-catenin in tumor cells. β-catenin at the invasive front of GCs is partly explained by interactions within the tumor microenvironment. We confirmed *in vitro* that macrophages increase the invasion ability of GC cells. Using immunofluorescence confocal microscopy, we determined localization and accumulation of β-catenin in the nucleus in treated macrophages. Thus, we suspect that cytokines secreted by macrophages induce β-catenin nuclear translocation in GC cells, thus increasing their invasion ability. The invasive front of epithelial tumors represents a microenvironment in which macrophages interact with parenchymal cells by producing extracellular matrix and by secreting cytokines and growth factors that locally promote cell proliferation and invasion.[26, 27] However, in our study, increased β-catenin expression *in vitro* did not alter proliferation, indicating that by itself, is not sufficient to augment tumor malignancy. This finding is also demonstrated previously that β-catenin only promotes adhesion sites to form focally and stimulates directional migration and invasion of colon cancer cells but not involved in the proliferation of gastric and prostate cancer cells.[28–30] Nevertheless, the stromal cell paracrine modulation of Wnt signaling in epithelial cells is likely coordinated by a more complex network of promoting and inhibiting secretion factors. The downstream effects of different levels of Wnt/β-catenin signaling (and in particular those affecting cell proliferation, EMT and local invasion) are as of yet poorly understood. β-catenin translocates to the nucleus where it binds to transcription factors of the T cell factor/lymphocyte enhancer factor (TCF/LEF) family and initiates transcription of target genes such as *cyclin D1*, *c-myc* and *MMP-7*[31, 32] involved in proliferation, tumor invasion and metastasis.[33, 34] Our findings suggest that the signalling pathways through which macrophages induce β-catenin also induce the expression of target genes. Consistent with our previous work,[5] MMP-7 and CD44 were two PCR arrays identified after coculture with macrophages as differentially expressed genes associated with metastasis of GC cells. Degradation of the extracellular matrix mediated by matrix metalloproteinases (MMPs) is a crucial mechanism during tumor invasion and metastasis. Such degradation is necessary to create a microenvironment that supports the growth of primary tumors and metastasis. The β-catenin/TCF complex subsequently activates a large number of oncogenes, including vegf, c-myc, c-jun, fra-1 and gastrin that may also be involved in macrophage activated β-catenin downstream events.[35]

A variety of routes likely modulate the Wnt/β-catenin pathway and regulation of β-catenin signaling may be similarly complicated. Recent studies found that macrophage CM triggers the ERK pathway and Wnt activates ERK in endothelial cells.[36, 37] Here, we found that macrophages induce phosphorylation of both ERK and AKT in GC cells. We demonstrated that treatment with AKT inhibitor but not ERK inhibitor reduces β-catenin translocation. These results indicate that Akt plays roles in macrophage activated β-catenin signaling in gastric cell types.

## Conclusions

Collectively, the clinical data suggest that macrophage infiltration is correlated with increased grade and poorer prognosis for GC patients who underwent radical resection. Macrophages may induce GC invasiveness by activating the β-catenin pathway. Accordingly, the suppression of macrophage infiltration and suppression of activation of the β-catenin pathway represent potential strategies for treatment of GC.

## Supporting Information

S1 Fig Effect of macrophage CM on β-catenin pathway. β-catenin accumulates in nucleus after treatment with macrophage CM for 30 minutes in AGS and MKN45 cells.
(TIFF)

S2 Fig Effect of macrophage CM on protein expression of β-catenin down-stream genes of AGS and MKN45 cells. (TIFF)

S3 Fig (A) Macrophage CM induced AKT and ERK activation in N87 cells. (B) N87 cells were pre-treated with 10 μM of the β-catenin inhibitors: LY294002 or U0126 for 1 hour then cultured in the presence of macrophage CM for 1 additional hour. AKT, ERK and β-catenin protein expression were determined by Western blot.
(TIFF)

S4 Fig Neutralizing antibody against TNF-α. N87 cells in the presence of macrophage CM for 24 hours were pre-treated with or without TNF-α inhibitor for 1 hour.
(TIFF)
