# Supplementary figures and images for: Macrophage Infiltration Induces Gastric Cancer Invasiveness by Activating the β-Catenin Pathway
Source: PLoS One. 2015 Jul 30;10(7):e0134122. doi: 10.1371/journal.pone.0134122 (PMC4520459; doi:10.1371/journal.pone.0134122)

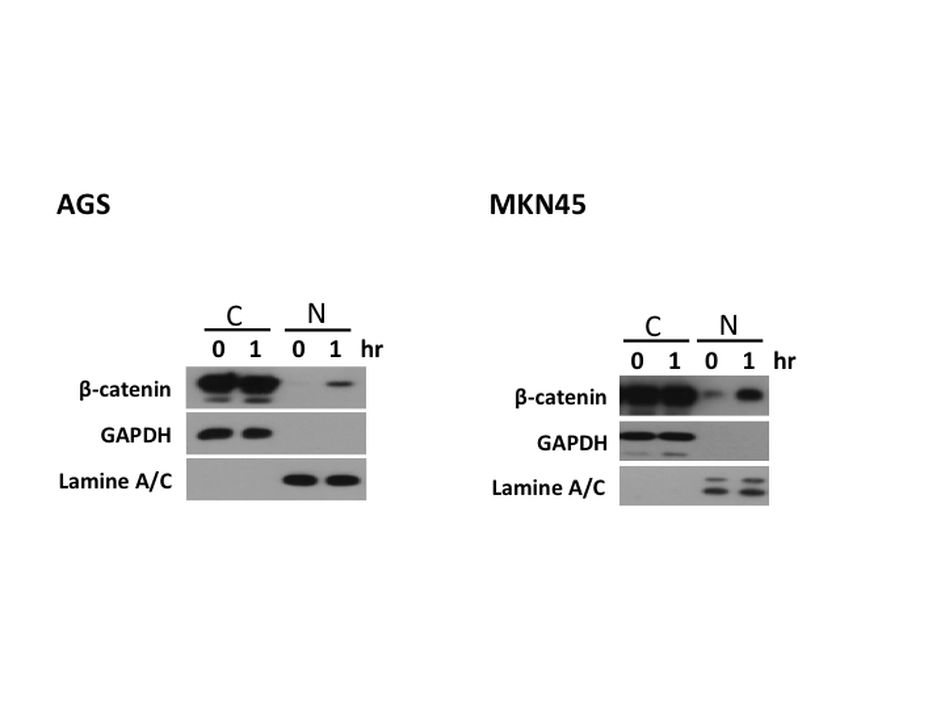

Supplement: S1 Fig — β-catenin accumulates in nucleus after treatment with macrophage CM for 30 minutes in AGS and MKN45 cells. (TIFF) [file pone.0134122.s001.tiff]

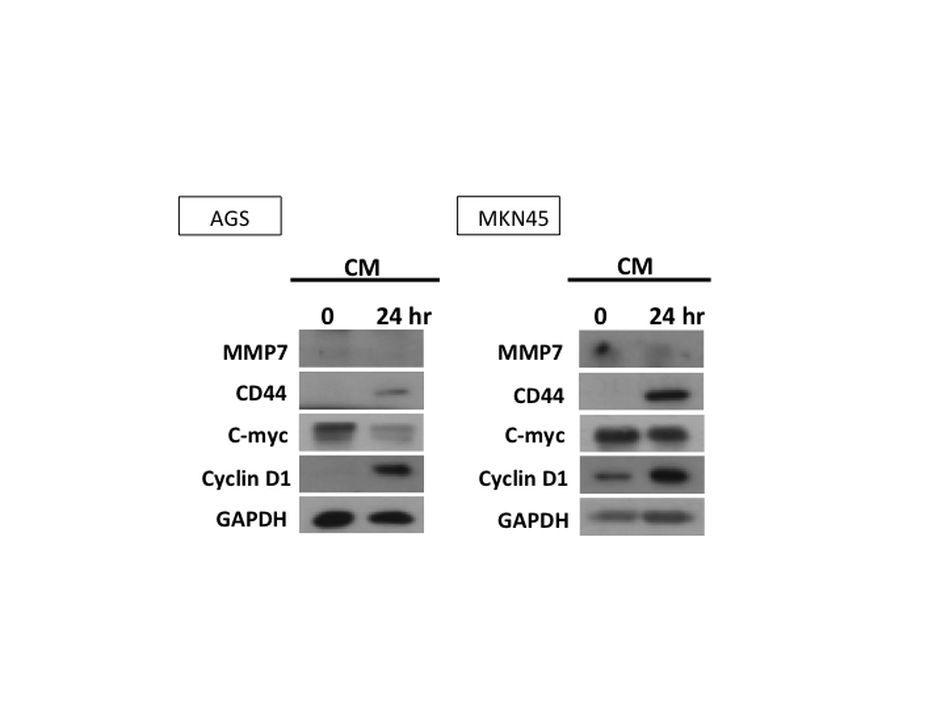

Supplement: S2 Fig — (TIFF) [file pone.0134122.s002.tiff]

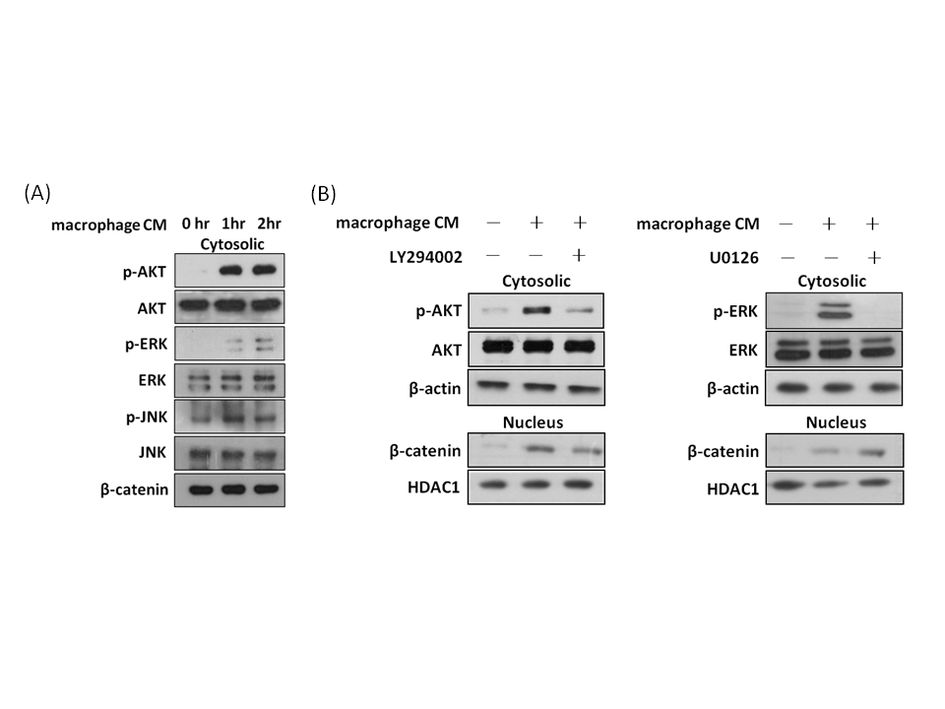

Supplement: S3 Fig — AKT, ERK and β-catenin protein expression were determined by Western blot. (TIFF) [file pone.0134122.s003.tiff]

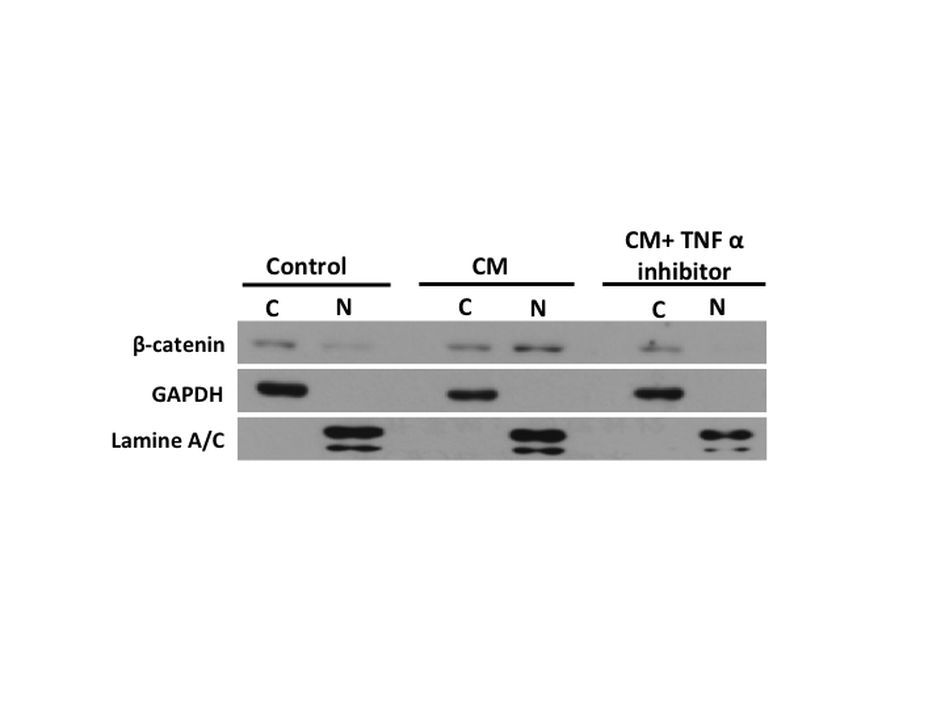

Supplement: S4 Fig — N87 cells in the presence of macrophage CM for 24 hours were pre-treated with or without TNF-α inhibitor for 1 hour. (TIFF) [file pone.0134122.s004.tiff]
